# WEBnm@ v2.0: Web server and services for comparing protein flexibility

**DOI:** 10.1186/s12859-014-0427-6

**Published:** 2014-12-30

**Authors:** Sandhya P Tiwari, Edvin Fuglebakk, Siv M Hollup, Lars Skjærven, Tristan Cragnolini, Svenn H Grindhaug, Kidane M Tekle, Nathalie Reuter

**Affiliations:** Department of Molecular Biology, University of Bergen, Bergen, Norway; Department of Biomedicine, University of Bergen, Bergen, Norway; Computational Biology Unit, Department of Informatics, University of Bergen, Bergen, Norway; Present address: University Chemical Laboratories, University of Cambridge, Lensfield Road, Cambridge, CB2 1EW UK

**Keywords:** Elastic network models, Normal mode analysis, Comparative analysis, Web-tool, TIM barrels, Adenylate Kinase, Bhattacharyya Coefficient

## Abstract

**Background:**

Normal mode analysis (NMA) using elastic network models is a reliable and cost-effective computational method to characterise protein flexibility and by extension, their dynamics. Further insight into the dynamics–function relationship can be gained by comparing protein motions between protein homologs and functional classifications. This can be achieved by comparing normal modes obtained from sets of evolutionary related proteins.

**Results:**

We have developed an automated tool for comparative NMA of a set of pre-aligned protein structures. The user can submit a sequence alignment in the FASTA format and the corresponding coordinate files in the Protein Data Bank (PDB) format. The computed normalised squared atomic fluctuations and atomic deformation energies of the submitted structures can be easily compared on graphs provided by the web user interface. The web server provides pairwise comparison of the dynamics of all proteins included in the submitted set using two measures: the Root Mean Squared Inner Product and the Bhattacharyya Coefficient. The Comparative Analysis has been implemented on our web server for NMA, WEBnm@, which also provides recently upgraded functionality for NMA of single protein structures. This includes new visualisations of protein motion, visualisation of inter-residue correlations and the analysis of conformational change using the *overlap analysis*. In addition, programmatic access to WEBnm@ is now available through a SOAP-based web service. Webnm@ is available at http://apps.cbu.uib.no/webnma.

**Conclusion:**

WEBnm@ v2.0 is an online tool offering unique capability for comparative NMA on multiple protein structures. Along with a convenient web interface, powerful computing resources, and several methods for mode analyses, WEBnm@ facilitates the assessment of protein flexibility within protein families and superfamilies. These analyses can give a good view of how the structures move and how the flexibility is conserved over the different structures.

**Electronic supplementary material:**

The online version of this article (doi:10.1186/s12859-014-0427-6) contains supplementary material, which is available to authorized users.

## Background

Protein dynamics is defined as the time-dependent changes in the structure of a protein, which includes equilibrium fluctuations governing biological function [1]. The internal deformations of protein structures have been used successfully to describe components of these time-dependent fluctuations. The mechanisms of protein function exploit their structural flexibility at all levels, from the localised fluctuations of amino acid side chains, to the reorientation of large rigid bodies such as protein domains. In the past, much of these studies have been limited to qualitative descriptions of a limited number of static structures, but there is growing evidence that structure is linked to function via dynamics and that these may be structurally and evolutionarily conserved. Thus, we see a need for comparing protein dynamics in a systematic, quantitative manner.

Although the quantity of dynamical experimental data is ever increasing e.g. from the introduction of the time dimension in X-ray and NMR studies, the information gained from them is still sparse. Several computational methods have been developed to complement experimental structural biology data and provide dynamical models of biomolecules. Molecular Dynamics (MD) simulations are widely used to simulate the dynamics of a protein on time scales of up to the microsecond. It remains computationally expensive to perform MD simulations and the time scales necessary to satisfactorily sample large motions such as domain reorientation are typically not feasible to obtain. Normal Mode Analysis (NMA) using Elastic Network Models (ENM) is a computationally efficient and reliable method for predicting and characterising slow motions of proteins. The efficiency of ENM approaches also makes them particularly well suited for automated comparative analysis between multiple structures.

NMA has been increasingly used to capture the inherent flexibility of proteins [2-5]. NMA models the motions of atoms in a protein as a coupled harmonic oscillator and associates each mode of motion to a frequency of oscillation. Low frequency modes correspond to global or domain motions and have been found to correlate well to functionally relevant protein motions [6-9]. Moreover it has been shown to yield results in good agreement with molecular dynamics simulations when characterising the collective motions of proteins [5,10-14]. In ENMs, the protein is represented as a network of springs capturing the density of particles and strength of interaction between them. Inter-atomic interactions are described by a simple harmonic potential and the protein is often modelled with a reduced number of atoms, typically one bead per residue located at the C-alpha position. This granularity of the model provides a well-tested reduction in dimensionality, motivated by the approximate independence of whole-residue motion from side-chain dynamics in globular proteins [15].

Comparative analysis is a promising approach for exploring the connection between internal dynamics and structural and functional similarities between protein structures. It has been used to validate using ENM-based NMA as a method and developing measures to compare protein homologues [16-23]. Moreover, the protein fold [24] determines some aspects of the similarity between internal dynamics, which have been shown to be robust with regards to mutations or other local perturbations [25-27]. Comparing homologous proteins, or even structural variants of identical proteins, can therefore provide a useful check for whether properties of protein intrinsic motions ascribed to the shape or fold of the protein generalises to other structures with similar fold. Some examples of successful applications include comparison of homologues to understand allostery [28,29], oligomerisation [30] and enzymatic mechanisms [31-34]. For more insights into such analyses refer to [35,36].

The efficiency of ENMs has motivated the development of several online tools providing the calculation and analyses of normal modes of a single protein structure [23,37-42]. However, studying comparative protein flexibility requires suitable functionality and interface for analyses on multiple structures. WEBnm@ [43] has served structural biologists in exploring and analysing the intrinsic flexibility of single protein structures for almost a decade [44-53]. Here, we present a new version of WEBnm@ with enhanced functionality to support the exploratory comparative analysis of sets of protein structures for the sufficiently advanced user. In addition to the new *Comparative Analysis*, the new web server still provides access to the tools for analysing single protein structures (the *Single Analysis*). The Comparative Analysis section of the web server includes three key analyses; the deformation energies, normalised fluctuation profiles, comparisons of the lower frequency modes (Root Mean Squared Inner Product, RMSIP) and covariance matrices (Bhattacharyya coefficient, BC) calculated from the normal modes. In addition, web services have been set up for programmatic access to WEBnm@ v2.0.

In WEBnm@ v2.0 we have upgraded the capabilities of the Single Analysis by adding Jmol-based animations for the six lowest energy modes, a tool for calculating and visualising correlation matrices and overlap analysis for relating observed conformational changes with the normal modes of the proteins. The Jmol [54] application allows the user to manipulate the animation and visualise the vector-field of the movements. A correlation matrix is calculated from all the modes to show correlated movements within the structure and this can also be visualised both as a heat map and in PyMOL [55] on the input structure via a generated script. The overlap analysis captures the modes that correspond to a conformational change of the same protein.

We demonstrate the usability of WEBnm@ v2.0 with two case studies: the first on the TIM barrel superfamilies and the second on the Adenylate Kinases. In the first example, we find that we are able to discriminate structures that are related at the family and superfamily levels using the BC measure. In the second example, we find that the comparative analysis can be used to study the ligand-binding effects on the Adenylate Kinase. In a set of 8 homologous structures with conserved domains yet different conformations, we were able to easily cluster these structures based on their state, and scrutinise their differences further at the residue-level. We were able to identify changes in flexibility in one of the domains that could be a key difference between the ligand-free and ligand-bound structures. The analysis was able to capture these differences without the explicit modelling of the ligands. These examples demonstrate the ease and reliability with which large-scale NMA analysis can be performed via WEBnm@ v2.0 and potential applications to studying a set of protein structures with varying levels of homology.

## Material and methods

### Coarse-grained normal modes calculations

WEBnm@ employs the Elastic Network Model (ENM) with the C-alpha force field developed by Hinsen *et al.* [15], available in the Molecular Modelling ToolKit (MMTK) [56]. Each amino acid is represented by a mass at the position of its Cα atom. The interaction between two Cα atoms is described by the pair potential,

$$ {U}_{ij}\left(\mathbf{R}\right)=k\left(\left\Vert {\mathbf{R}}_{ij}^0\right\Vert \right){\left(\left\Vert {\mathbf{R}}_{ij}\right\Vert -\left\Vert {\mathbf{R}}_{ij}^0\right\Vert \right)}^2 $$

where:

$$ k(r)=\left\{\begin{array}{c}\hfill ar-b,\kern0.5em \mathrm{f}\mathrm{o}\mathrm{r}\kern0.5em r\kern0.5em <\kern0.5em d,\hfill \\ {}\hfill c{r}^{-6},\kern0.5em \mathrm{f}\mathrm{o}\mathrm{r}\kern0.5em r\kern0.5em \ge \kern0.5em d.\kern0.5em \hfill \end{array}\right\} $$

The following parameters for the force constant have been determined by Hinsen *et al.*, fitting to an all-atom model [15]: a = 8.6x10^5^ kJ mol^−1^ nm^−3^; b = 2.39x10^5^ kJ mol^−1^ nm^2^; c = 128 kJ mol^−1^ nm^4^ and d = 0.4 nm. Here, ***R***_***ij***_ is the pair distance vector between two Cα atoms and ***R***_***ij***_^***0***^ is the corresponding pair distance vector in the input configuration. Since the distance between Cα atoms adjacent in sequence clusters just short of 0.4 nm in typical protein structures, the potential can be regarded as having almost uniform force-constants for these interactions, with other interactions proportional to an inverse power of six of the equilibrium distance between interacting atoms. We do not provide an interface for adjusting these parameters as the parameterisation has been found to be transferable between proteins [15]. We prefer this approach to less detailed models that require parameterisation for each protein. Protein specific parameterisation is a concern, because data for validation is scarcely available and one usually has to resort to parameterise against crystallographic B-factors, which is highly disputed as a model of thermal fluctuations [12,57-59].

The potential energy of a configuration **R** of the ENM is then:

$$ U\left(\mathbf{R}\right)={\displaystyle \sum_{\mathrm{all}\kern0.5em \mathrm{pairs}\kern0.5em \mathrm{i},\mathrm{j}}{U}_{ij}\left(\mathbf{R}\right)} $$

The normal modes are eigenvectors of the mass weighted matrix of second partial derivatives of the potential U. They describe deformations intrinsic to the protein structure. The eigenvalues correspond to the squares of the frequencies for each mode.

In the comparative analysis, the full set of modes is calculated for each structure submitted. In the single analysis, a method approximates the normal modes using a smaller basis set, which reduces the dimension of the input structure for efficient computation [60]. In cases where the structure is less than 300 Cα atoms or when convergence is not reached for larger structures, a complete basis set is used. All the analyses performed under single analysis, regardless of the size of the basis set, use only the first 200 non-trivial modes calculated. The vectors of these 200 modes are available for download in a text file. To ensure that the potential as defined above is minimal at the input structure, any anomalous distances shorter than 2.78 Å between two Cα atoms raise an error, this distance being defined by the parameters used in the potential described above.

### Comparative analysis

#### Profile alignment analysis

Fluctuations are calculated for each Cα atom from the obtained set of modes calculated. These can be described as the sum of each atom’s displacement along each mode (excluding the trivial ones), weighted by the reciprocal of the eigenvalues. The deformation energy is a normalised measure of the energy contributed from individual atoms of the model to deformations of the structure (cf. [60] for more details). Low deformation energies may signify the presence of a rigid domain, while the presence of large deformation energies between rigid domains may signify the presence of a flexible hinge. Both the fluctuations and the deformation energy values reported are averaged over all modes and they are tabulated following the sequence alignment so that the results are ready for comparison purposes. The corresponding plot is generated using R [61] and made available on the web server as a PDF file, along with the raw data values as a text file.

#### Covariance similarity analysis

For comparing large sets of aligned protein structures, it is useful to obtain a single score characterising the level of similarity in the intrinsic motion. We implemented two measures for that purpose: the root mean squared inner product (RMSIP) [62] and the Bhattacharyya coefficient (BC) [21]. Both measures are calculated on amino acids that are conserved in the alignment of the whole dataset. Normal modes for only the conserved part of the alignment, which is derived from the following:

$$ \overset{..}{\mathbf{H}}={\mathbf{H}}_{aa}-{\mathbf{H}}_{ab}{\mathbf{H}}_{bb}^{-1}{\mathbf{H}}_{ab}^{\mathrm{T}} $$

where the Hessian (**H**) of the full potential is partitioned so that **H**_*aa*_ reflect interactions in conserved parts of the alignment, *a*, **H**_*ab*_ reflect interactions between *a* and non-conserved parts, *b*, and **H**_*bb*_ reflect interactions in *b*.

The results are presented as a heat map with a dendrogram obtained from a complete linkage clustering. This can be used to check whether the similarity of normal modes agree with functional or evolutionary classifications. The root mean squared inner product (RMSIP) is defined as:

$$ RMSIP={\left(\frac{1}{10}\left[{\displaystyle \sum_{i=1}^{10}{\displaystyle \sum_{j=1}^{10}{\left({\mathbf{X}}_i\cdot {\mathbf{Y}}_j\right)}^2}}\right]\right)}^{\frac{1}{2}} $$

where **X**_**i**_ and **Y**_**j**_ refer to the eigenvectors of a pair of proteins being compared, and i,j are the mode numbers. Following Amadei *et al.* [63] it is customary to let the sums run over the ten lowest energy non-trivial modes. The BC measure is based on the Bhattacharyya distance [64] that we adapted earlier for the purpose of comparing protein flexibility [21]. It compares the covariance matrices $\left(\overset{\sim }{\mathbf{A}}\kern0.5em \mathrm{and}\kern0.5em \overset{\sim }{\mathbf{B}}\right)$ obtained from the normal modes of the conserved parts of the proteins to be compared.

$$ BC= \exp \left(-\frac{1}{2s}1\mathrm{n}\left[\left|\frac{1}{2}\left(\overset{\sim }{\mathbf{A}}+\overset{\sim }{\mathbf{B}}\right)\right|{\left(\left|\overset{\sim }{\mathbf{A}}\right|\left|\overset{\sim }{\mathbf{B}}\right|\right)}^{-\frac{1}{2}}\right]\right) $$

Here |**X**| denotes the determinant of **X** and the rank of the matrices are reduced in two steps. First, **A**_**n**_ and **B**_**m**_ are obtained from the *n* and *m* lowest frequency modes of their respective proteins and normalised by dividing by their trace. Then $\left(\overset{\sim }{\mathbf{A}}\kern0.5em \mathrm{and}\kern0.5em \overset{\sim }{\mathbf{B}}\right)$ are obtained by projecting **A**_**n**_ and **B**_**m**_ on to the *s* eigenvectors of (**A**_**n**_ + **B**_**m**_)/2 that explain most of its variance. For each comparison *n* and *m* are chosen so that 95% of the variance of each protein is retained and *s* so that 75% of the variance of (**A**_**n**_ + **B**_**m**_)/2 is retained. The initial rank reduction, obtained by **A**_**n**_ and **B**_**m**_ is introduced in the web server for computational efficiency.

### Single analysis

#### Mode Visualization

Animations of the six lowest frequency modes are provided in the web user interface through a Jmol applet. They display vector field arrows, which show the magnitude and direction of the motions characterised by each mode. The Jmol applet takes standard Jmol commands for modifying the visual representation, and thereby allowing e.g. change of representation, distance measurements, etc. All of the modes eigenvectors and eigenvalues are also available as text files for further manipulation by a more advanced user.

#### Correlation matrix analysis

The correlation matrix as defined by Ichiye and Karplus [65] is calculated from the normal modes. Each element in the matrix quantifies the coupling between two atoms i and j as:

$$ {\mathrm{C}}_{ij}=\frac{{\displaystyle {\sum}_{m=1}^M\frac{1}{\lambda_m}{\left[{\mathrm{X}}_m\right]}_i\cdot }{\left[{\mathrm{X}}_m\right]}_j}{{\left({\displaystyle {\sum}_{m=1}^M\frac{1}{\lambda_m}{\left[{\mathrm{X}}_m\right]}_i\cdot }{\left[{\mathrm{X}}_m\right]}_i\right)}^{\frac{1}{2}}{\left({\displaystyle {\sum}_{m=1}^M\frac{1}{\lambda_m}{\left[{\mathrm{X}}_m\right]}_j\cdot }{\left[{\mathrm{X}}_m\right]}_j\right)}^{\frac{1}{2}}} $$

where **X**_m_ and λ_m_ are eigenvectors and eigenvalues of the *m*^th^ normal mode respectively and the i and j indices denote the component of the mode corresponding to individual atoms. C_ij_ is the expected inner product of displacements of atoms i and j, and ranges from −1 to 1, where −1 and 1 are maximal anti-correlations and correlations.

A visual representation of these correlated regions is available as a downloadable PyMOL script, where significant correlations are represented as sticks on the cartoon representation of the structure. These correlations are chosen such that:

- They are above the chosen threshold for positive correlations (represented as red sticks), and below the negative of this threshold for the negative correlations (represented as blue sticks). The correlation threshold is picked at a percentile that changes according to the number of Cα atoms in the input structure; the percentile lies within the range of 95, for less than 200 atoms, to 99.9, for more than 2000 atoms. The percentile chosen for a given structure and the resulting threshold values are provided as a header comments in the downloadable PyMOL script.
- Only the correlations between atoms that have a minimum distance of 0.8 nm are considered, to focus on the pairs of Cα atoms that have a limited influence from the peptide backbone and strong force constants in the ENM.
- The network of residues that satisfies the score threshold has a minimum size of 1 pair of Cα atoms.

Based on the above criteria, the visual representation is especially informative with structures that possess more than one domain.

#### Overlap analysis

The overlap analysis compares two conformations of the same protein (e.g. A and B), submitted by the user, and identifies the modes that contribute the most to the structural difference; these modes are likely to be involved in the movements leading from conformation A to B [66]. The web server calculates the overlap between the modes calculated and the structural difference between the two submitted structures. Values of the squared overlap and the cumulative overlap are plotted against mode numbers.

## Implementation

The Webnm@ back-end is implemented using the Molecular Modelling Toolkit (MMTK) [56] and runs on a dedicated server currently utilising 24 2.4 GHz cores with 256GB of available memory. The Application Programming Interface (API) is written in Python and the web interface is built on the Zope ToolKit [67] - based Grok framework 1.0 [68]. Management of multiple jobs is taken care of by an in-house job scheduler.

### Web services

We have developed two web services, one for the single analysis and one for the comparative analysis. Both are implemented as document/literal wrapped SOAP [69] web-services. The web-services provide only the raw data points as output and exclude the animation files. WSDL files (Web Service Definition Language) for accessing document/literal wrapped SOAP web services are available at http://cbu.bioinfo.no/wsdl/webnma-single.wsdl (Single Analysis) and http://cbu.bioinfo.no/wsdl/webnma-comparative.wsdl (Comparative Analysis). Example scripts detailing the use of the services can be found on the website.

## Results and discussion

### Description of the web server

For protein structures, WEBnm@ requires Cα coordinates as input, which it gathers from a PDB ID or coordinate file. Users are provided with a unique URL, or alternatively to provide their email address to receive the link that would allow the user to retrieve their results upon convenience (within two weeks of the submission date). On the WEBnm@ v2.0 page, a user is able to choose between Single Analysis and Comparative Analysis through two different tabs.

**Figure 1:**
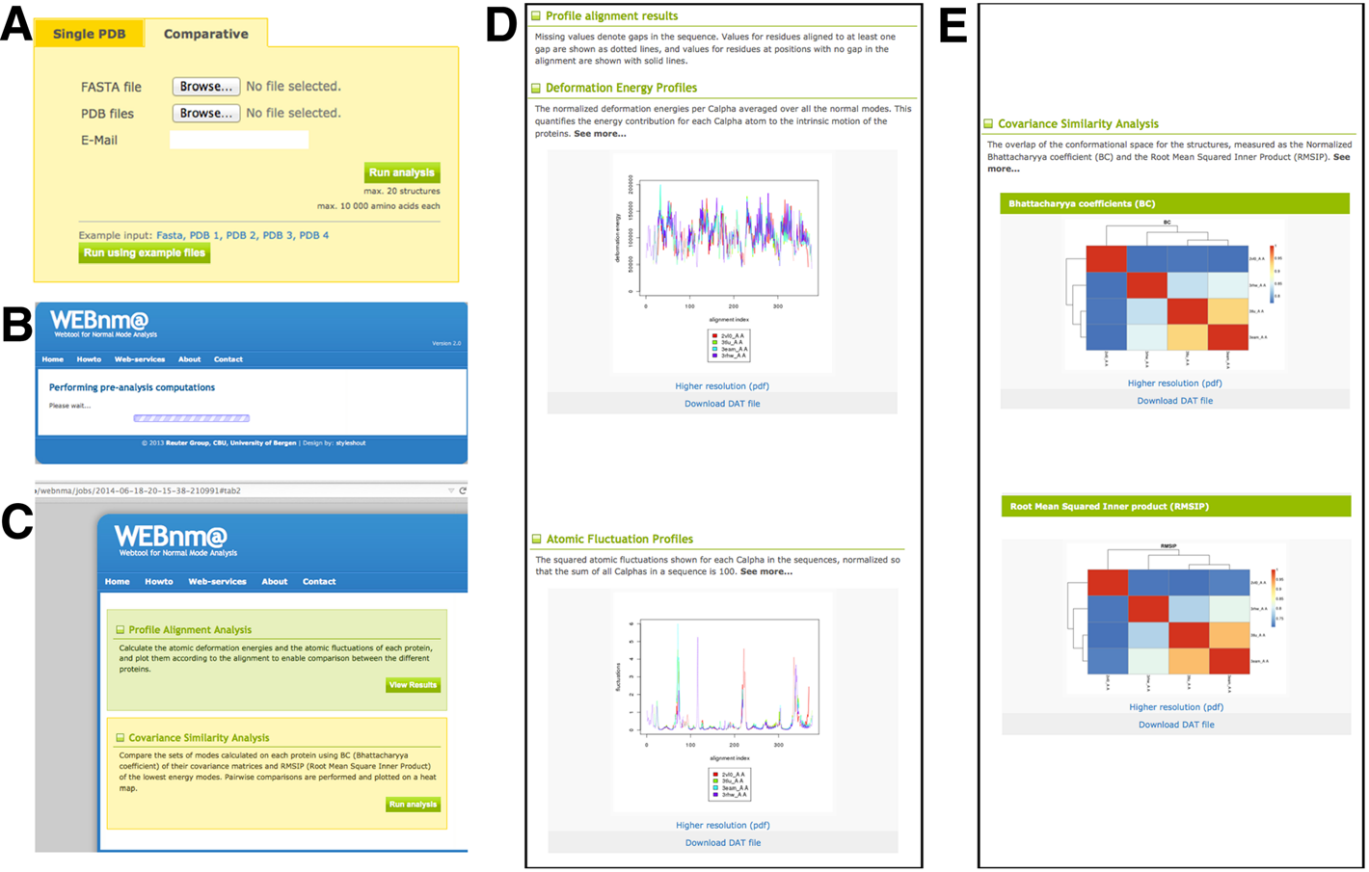
**Flow-chart of the webUI. (A)** File upload and job submission, **(B)** modes computation and preliminary analysis, **(C)** list of analyses available, **(D)** profile alignment results, **(E)** covariance similarity analysis.

A flowchart illustrating the steps and functionality of the Comparative Analysis server is provided on Figure 1 and the main steps are described here:

A) The input for the comparative analysis can be submitted under the ‘Comparative’ tab, where an alignment in the sequence-based FASTA format can be uploaded under the ‘FASTA file’ field, and the corresponding coordinate files in the ‘PDB files’ field. Although the alignment format requires alignments to respect sequential order, the alignments should preferably be obtained from a structural alignment algorithm as they have been shown to be more reliable when comparing structures and their intrinsic dynamics [70]. The sequence-based FASTA format of the alignment is provided by many popular structural alignment algorithms. We provide format description and examples on the web user interface (webUI), as well as advice on how to best align the structures. An e-mail address can be provided (but is not required) for larger jobs so that the user can receive the URL to his/her results.
B) After the initial submission, all jobs go through a pre-analysis computation phase where the normal modes are calculated and initial analyses are performed. From this stage on, users can bookmark the URL that will allow them to retrieve their results at their convenience, within two weeks of the submission date.
C) The user can choose the appropriate analysis to be performed: profile alignment analysis for deformation energies and atomic fluctuations, and covariance similarity analysis for calculations of the RMSIP and BC. Warnings appear (red fonts) if the structure(s) submitted contain unrecognised heteroatoms or non-standard amino acids to inform the user how these have been taken into account in the modes calculation. From this point onwards, brief descriptions and references for the results provided upon expanding the “See more…” button in the results pages.
D) The ‘Profile Alignment Analysis’ results in a plot of the deformation energy for each protein in the dataset with respect to the sequence number in the alignment. The same format is used for the atomic fluctuation profiles. The results are also provided as raw data (text files, with no values set for positions corresponding to gaps in the alignment).
E) The ‘Covariance Similarity Analysis’ provides as output BC and RMSIP heat maps. Links to their full PDF versions at higher resolution are provided as well as the raw data should the users wish to produce their own plots.

### Case study 1: TIM-barrel proteins fold

**Figure 2:**
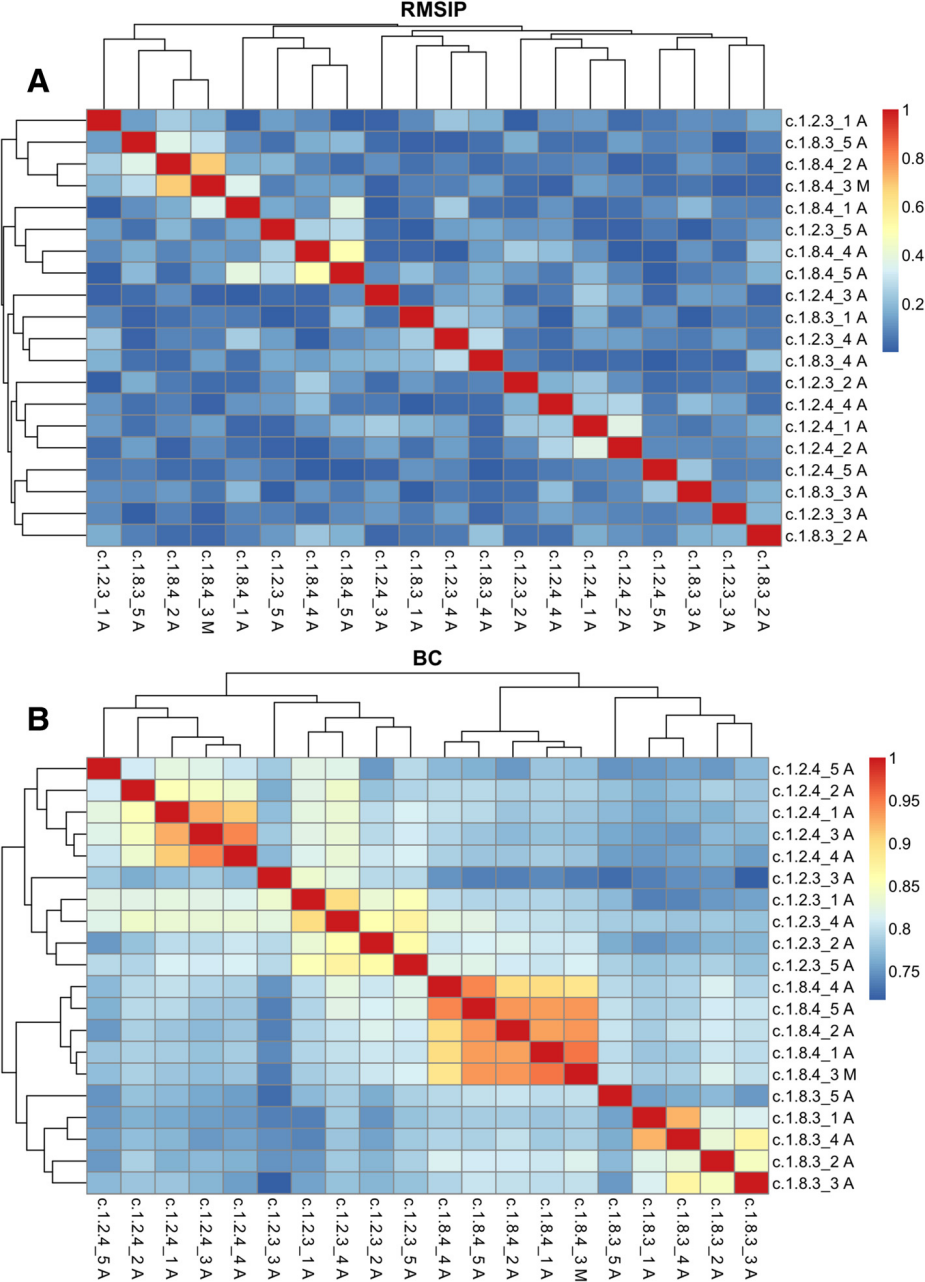
**Root Mean Squared Inner Product (RMSIP, A) and the Bhattacharyya Coefficient (BC, B) for all pairs of structures in the TIM beta/alpha-barrel fold.** The plots are symmetric with comparisons of a structure to itself on the diagonal and hierarchical clustering represented on the periphery. The colour scale is adapted to visualise contrasts in a data set, hence the same colour might refer to different values in the two plots. Structure names reflect the SCOP classification, with leading characters denoting the SCOP concise classification string.

We have previously studied the conservation of intrinsic protein motions by comparing the normal modes between diverged protein structures [21]. We present here an example of the same type of study as exploratory analysis. We aligned 20 structures from the TIM beta/alpha-barrel fold as classified by SCOP [71] using MUSTANG [72]. MUSTANG aligns the Cα atoms of proteins using a progressive pairwise framework that is later optimised in the context of all the structures in the alignment. It is able to align the structures based on the similarity of their residue-residue contacts and local structural topology and has been shown to be one of the top performing methods [73]. The structures were selected such that two superfamilies are equally represented, and from each of these two families are equally represented. We then submitted this alignment and the corresponding protein structures to the web server, which calculated the similarity of the normal modes for all pairs of structures in the data set. Having ordered the alignment file by family and superfamily the resulting plots are presented in Figure 2. We report both RMSIP (Figure 2A) and BC (Figure 2B). It can be seen at a glance that the BC similarity measure discriminates between both families and superfamilies. Here, the dendrogram on one side matches the SCOP classification on the other. With the RMSIP, this classification is not captured, especially at the superfamily level. Comparing close and more remote homologues simultaneously provides the user an indication of how large cross-family and cross-superfamily comparisons need to be in order to be interpretable for a certain alignment. In general, groups of similar and less similar proteins can be obtained from classifications, by phylogenetic analysis or by distance measure comparing structure coordinates, such as the Root Mean Squared Deviation (RMSD). Explicitly inspecting that the distances between structures are also observed in terms of structural deformability, as assessed by the BC, serves to confirm that such structural differences are also reflected in the intrinsic dynamics. This example also demonstrates the use of explicitly factoring in the energetic separation between the modes compared, as is done with the BC, but not with the RMSIP. The BC, RMSD and RMSIP are compared in Fuglebakk *et al.* [21], and the sensitivity of such analysis to the quality of alignments are discussed in a recent review [70]. Figure 2B also indicates the selection of structures that are least similar to other structures sharing its classification. This illustrates how this analysis can be used to choose representative structures of a class for a more detailed study.

We believe that this type of analysis is useful for exploring the conservation of protein intrinsic motions and to check assumptions about the variability of such motions in data sets [16,30,32,34,74,75]. Other potentially useful applications could be contrasting isolated subunits with subunits in a protein complex [29], or proteins with and without a bound ligand [28,31,33]. For more detailed characterisation of the structural flexibility of any class of proteins, the visualisation in Figure 2 can be used to identify good representatives of the class for further investigation.

### Case study 2: Adenylate Kinases

The comparative analysis is also useful for analysing conformational changes in identical and distantly related orthologues. We describe here the example of Adenylate Kinases (AdKs), which are a well-studied family of enzymes that transfers a phosphoryl group from an ATP to an AMP to create 2 ADP molecules with the aid of a magnesium ion. These enzymes are considered to be critical in the regulation of diverse cellular processes such as metabolic monitory and cell signalling (for a full review: Zhang *et al.* [76]). These monomeric enzymes consist of a CORE domain and two flanking mobile domains called LID (which is the site of ATP-binding) and NMP-binding (which is the site of the AMP-binding). The conformational changes that occur upon substrate binding involve large-amplitude, hinge-like movements of the LID and NMP-binding domains, which falls within the nanosecond timescale [77-81]. These domains are also known to move in a correlated manner [82] where the closing and opening of the NMP-binding domain is enhanced by the closing and opening of the LID domain. Some studies have been suggesting that the domains also undergo partial unfolding as part of this process [83,84], whereas Daily *et al.* [85], amongst others, have suggested that many local motions are involved in the large conformational change seen.

**Figure 3:**
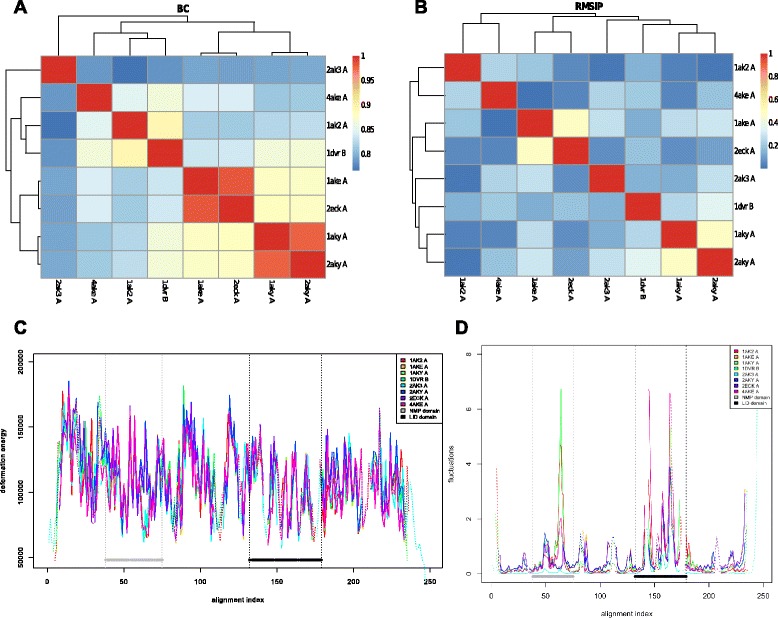
**Comparative analyses of the Adenylate Kinases.** The Bhattacharya Coefficient (BC) for all pairwise comparison in the alignment of the Adenylate Kinases **(A)**. The plot shows that the ligand-bound and ligand-free structures cluster in two distinct groups. The clustering obtained with RMSIP is also shown **(B)**. The deformation energy plot **(C)** and the comparative normalised fluctuations plot **(D) **show the values of the parts that are aligned to all other structures in the alignment as solid lines, and the parts aligned with gaps in other structures as dotted lines. The NMP and LID domains are marked by black and grey points, respectively.

We analysed AdKs in varying states, bound to different ligands (Table 1). Their sequences vary in their similarity, ranging from 52% to 100% (Additional file 1: Table S2). The states include fully open and ligand-free, partially open and ligand-free, NMP-binding domain closed (AMP bound), LID domain closed (ATP bound), both closed (AMP, ADP bound or AP5 bound with or without cofactor). For all of these structures, the calculations were done on only the Cα atoms of the structures, disregarding the presence of the ligands. Nevertheless, since the backbones of these structures are influenced by the conformation they are in, the spatial arrangement of these atoms is captured by their respective ENMs. We report here the BC scores for the 8 enzymes (Figure 3A), which show clear separation between the states of the enzymes, with the clustering showing the relative closeness of these states to each other. The separation between these structures follows that of the clustering done by Snow *et al.* [77] based on essential dynamics sampling simulations. The same clustering of the structures was not seen when comparing their RMSIP scores (Figure 3B). We see greater variation in the scores between the ligand-free structures than we do for the ligand-bound structures. This difference could correspond to the range of mobility of the hinge domains between the homologues that results in the fully and partially open sub-states. The outlier 2ak3 displays the same kind of order in the clustering with regards to 4ake, 1dvr and 1ake as seen in the separation of the distribution of LID-CORE and NMP-binding-CORE distances observed in all-atom MD simulations [86].

**Table 1:** **PDB IDs, chains, conformational state and ligands bound of the Adenylate Kinase analysed using comparative analysis in case study 2**

**PDB ID**	**Chain**	**State**	**Organism**	**Ligands**
2ak3	A	NMP-binding domain closed	*Bos taurus*	AMP
4ake	A	Both NMP-binding and LID domains open	*E. coli*	None
1ak2	A	LID domain nearly closed	*Bos taurus*	None
1dvr	B	LID domain closed	*S. cerevisiae*	ATP
1ake	A	Both NMP-binding and LID domains closed	*E. coli*	AP5
2eck	A	Both NMP-binding and LID domains closed	*E. coli*	AMP and ADP
1aky	A	Both NMP-binding and LID domains closed	*S. cerevisiae*	AP5 and imidazole
2aky	A	Both NMP-binding and LID domains closed	*S. cerevisiae*	AP5 and Mg2+

The BC score provides a measure of the similarity between the intrinsic motions of the proteins, but does not provide information about which parts of the structures accounts for any difference in intrinsic motion. To aid in pinpointing specific differences between them, the web server provides deformation energies and fluctuations calculated for each amino acid. We analysed the deformation energies and the fluctuations of these 8 structures (Figure 3C and D). On these plots, the fluctuations of the conserved part are represented using solid lines, and the non-conserved parts in dotted lines. In the comparative fluctuations plot, we find that the CORE domain between the LID and NMP-binding domains has higher relative flexibility in the structures with both domains closed (1ake, 1aky, 2aky, 2eck) than with the others. The structures that have the NMP-binding domain free of any ligand (4ake, 1ak2, 1dvr) display larger peaks within this domain, especially towards the C-terminal end, whereas the structures with ligands in this domain display low levels of fluctuations in contrast. While this trend is not as explicit in the LID domain, we do see that the fully open structure 4ake experiences the most fluctuations in this domain.

**Figure 4:**
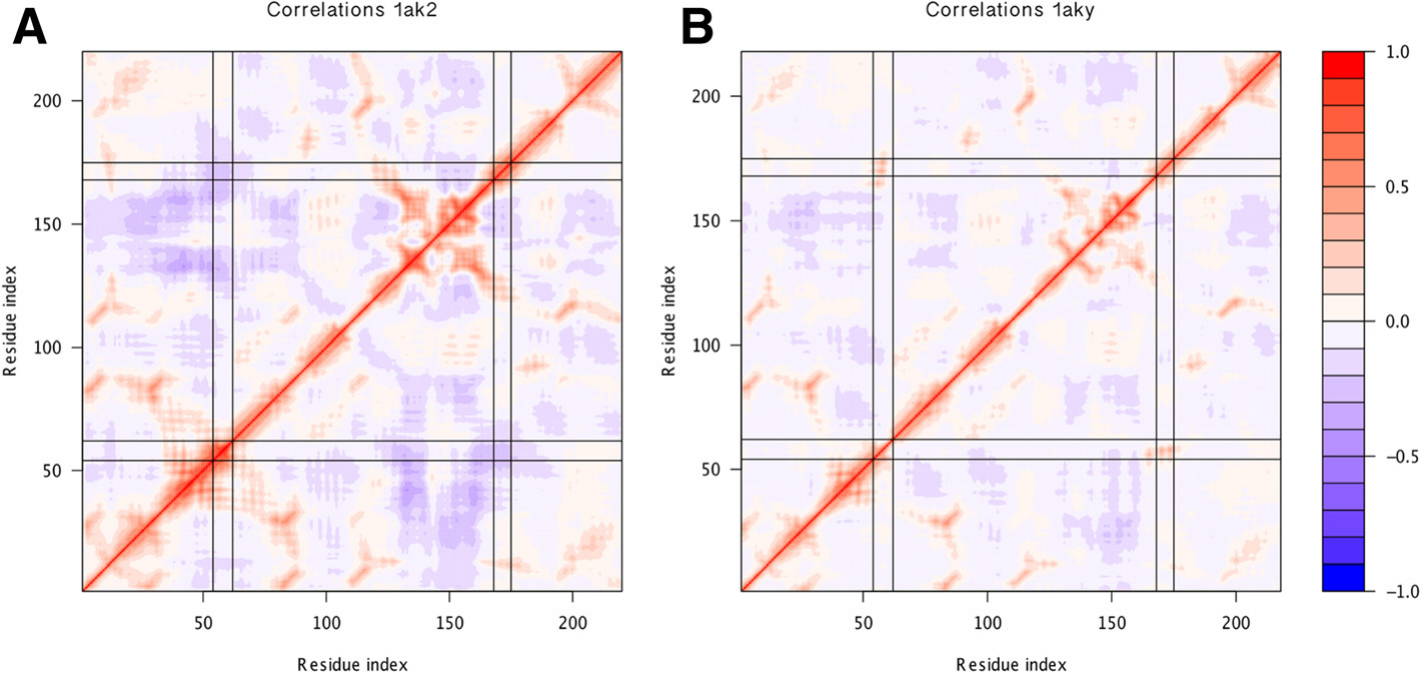
**Inter-residue correlations for selected representatives of the open and closed conformation of the Adenylate Kinases.** The open conformation **(A)** is represented by PDB ID 1ak2 and the closed conformation **(B)** by PDB ID 1aky. Solid black lines mark the regions that have greater correlations in the closed conformation than in the open (residue index 54–62 and 168–175).

In the comparative deformation energies plot, we see that structures that have no ligands or a ligand bound in only one of the domains display a clear opposite trend (a valley instead of a peak) towards the C-terminal end of the NMP-binding domain (positions 60–67 in the alignment index) when compared to structures that have both domains occupied. Upon closer scrutiny on the structure, this region lies in the loop between two helices within the domain that interacts with the ligand. The regions that flank this have been identified to be hinges that facilitate the displacement of the NMP-binding domain that have been described by Henzler-Wildman *et al.* [78], and fits well with the hinge region predicted by Pontiggia *et al.* [87]. This region also precedes the part of the NMP-binding domain that accumulates very high strain energy [88]. We observed that this region consists of higher deformation energies when both domains are bound, compared to the structures that have only one or neither of the domains occupied. The contrast in deformation energies in the NMP binding domain, fits well with the clusters obtained from the BC score, and suggests a local structural clue to what causes the difference in conformational degrees of freedom (seen in the BC plot) for these two groups of structures. Based on the BC plot we chose 1aky and 1ak2 as representatives of the closed and open or partially open conformation respectively. Inspection of these structures reveals that the region of the NMP domain with higher deformation energies in the closed state is in close contact with a helix flanking the LID domain. From the comparative fluctuations plot (Figure 3D), we see that both sides of this contact (alignment index 60–67 and 180–187) have increased fluctuations in the open states. Running 1aky and 1ak2 on the single analysis reveals correlation plots with a marked difference in correlation between these two regions. Compare the correlations between the segments with residue index 54–62 and 168–175 in Figure 4A and B marked by solid black lines. These regions in the closed conformation are highly positively correlated (Figure 4B), which is typical for parts of the structure in close contact. While we will be careful in making functional inference from these structural observations, we believe this example serves to illustrate how the comparison of fluctuation and deformation profiles can serve to localise structural features that can explain why the normal modes of related structures are different, as inferred from the BC or RMSIP plots. We have also illustrated how the single analysis on representative structures can aid in such structural inference.

This also shows that despite the lack of ligands represented explicitly in the ENMs, the analysis is still sensitive enough to pick up on changes in flexibility conferred to these points, even when the effects are small. Moreover, the heterogeneity in the conformations of the proteins can be well assessed by the similarity scoring (BC) and further scrutinised using the profile alignment techniques. The implication of comparing multiple structures, instead of the pair of completely open and closed structures, is that it allows us to understand the dynamics of intermediate conformational states of an enzyme in relation to each other, without over-interpreting differences between the two extreme cases.

### Elastic network models for comparative analysis

In general, we find ENMs to be an ideal tool for comparing structure-encoded dynamics in protein structures. Their robust parameterisation and computational convenience allows for rapid comparison of multiple structures and for interpretation in relatively few dimensions. Recent reviews on comparative analysis of protein internal dynamics have explicated the use of ENMs much further (cf. [36,70]). Despite some limitations of these models that should be taken into consideration by a user, we stress that ENMs are well suited to explore the structural degrees of freedom intrinsic to a proteins fold. The modulation of protein dynamics that is not mediated through changes in shape or local density of the protein should be addressed through other methods.

## Conclusion

WEBnm@ version 2.0 now provides comparative NMA on multiple protein structures, in addition to its original functionality as a web-tool for NMA performed on the ENMs of single protein structures. The computations are performed using the Cα force field developed by Hinsen *et al.* [15]. Comparative analyses of aligned structures are to the best of our knowledge not provided by any other web-tools. We have demonstrated that comparative analysis can be used to conduct analysis of evolutionarily related protein families, and study a small set of homologous proteins where the results may provide clues to the differences in flexibility. WEBnm@ is designed to quickly provide simple output, tailored towards teaching and exploratory analysis of proteins while large scale analysis can be accomplished through the web services.

## Availability and requirements

**Project name:** Webnm@

**Project home page:**http://apps.cbu.uib.no/webnma.

**Web services:**http://cbu.bioinfo.no/wsdl/webnma-single.wsdl, http://cbu.bioinfo.no/wsdl/webnma-comparative.wsdl

**Operating system(s):** Platform independent.

**Programming language:** Java and Javascript is required to utilise part of the interface. For programatic access, some functionality as document/literal wrapped SOAP web services.

**Other requirements:** Some visualisations require Java applets not signed by commercial authorities.

**License:** The webserver and web services are provided free of charge, with some limitations on the volume of analysis, which is described on the project home page. Source code is not available online.

**Any restrictions to use by non-academics:** None

## Additional file

Additional file 1: Figure S1. Flow of the Single Analysis on the webUI. **Figure S2.** Sequence representation of the structural alignment of the homologous Adenylate Kinases. **Table S1.** TIM barrel proteins fold dataset. **Table S2.** Sequence similarities of the homologous Adenylate Kinases sequences.

## Abbreviations

NMA: Normal mode analysis
ENM: Elastic network model
BC: Bhattacharyya coefficient
RMSIP: Root mean squared inner product
MD: Molecular dynamics
MMTK: Molecular modelling toolkit
