# Supplementary material for: WEBnm@ v2.0: Web server and services for comparing protein flexibility
Source: BMC Bioinformatics. 2014 Dec 30;15(1):427. doi: 10.1186/s12859-014-0427-6 (PMC4339738; doi:10.1186/s12859-014-0427-6)
Supplement: Additional file 1: Figure S1. — Flow of the Single Analysis on the webUI. Figure S2. Sequence representation of the structural alignment of the homologous Adenylate Kinases. Table S1. TIM barrel proteins fold dataset. Table S2. Sequence similarities of the homologous Adenylate Kinases sequences. [file 12859_2014_427_MOESM1_ESM.pdf]

WEBnm@ v2.0:
Web server and web services for comparing protein flexibility

Sandhya P. Tiwari [2;4], Edvin Fuglebakk [1;4], Siv M.Hollup [1;4], Lars Skjaerven [3;4], Tristan Cragnolini [2;4], Svenn H.Grindhaug [4], Kidane M. Tekle[4] and Nathalie Reuter* [2;41]

[1]Department of Informatics, University of Bergen, Norway,
[2]Department of Molecular Biology, University of Bergen, Norway,
[3]Department of Biomedicine, University of Bergen, Norway,
[4]Computational Biology Unit, Department of Informatics, University of Bergen, Norway

Supplementary Material

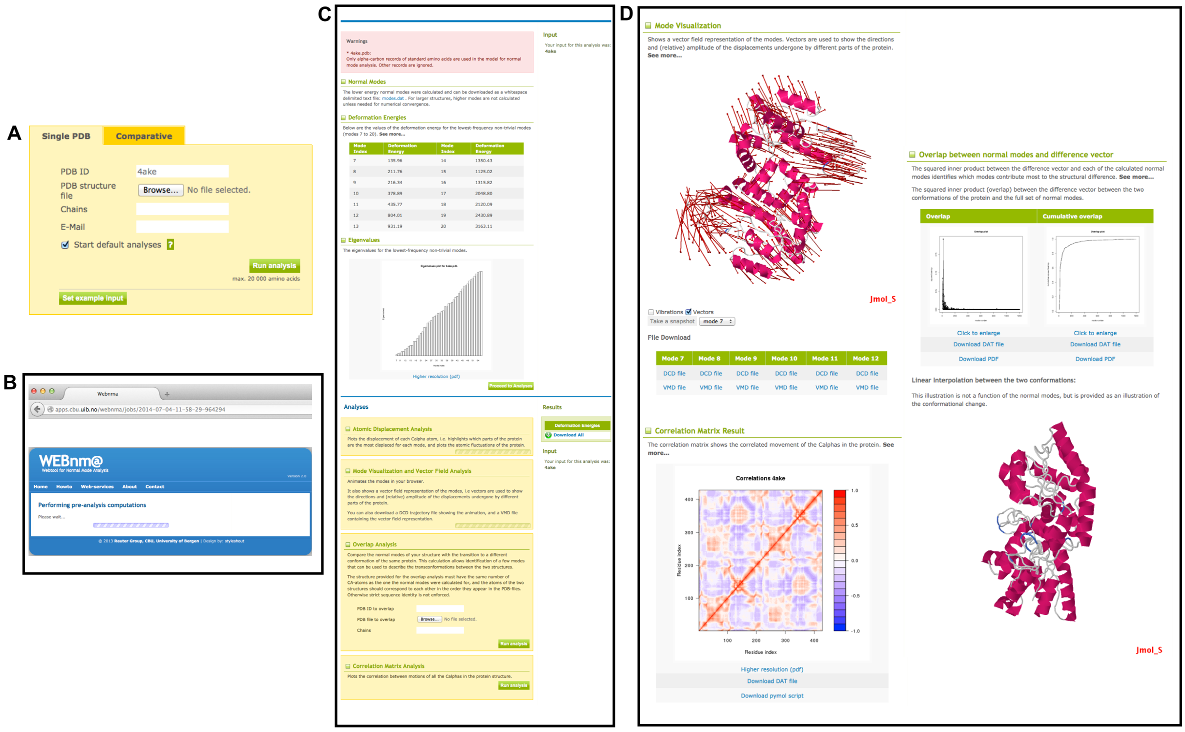

Figure S1. Flow of the Single Analysis on the webUI. A) The input, in the form of a PDB ID or a coordinate file, can be submitted in the ‘Single PDB’ tab. The user can also specify chain(s) in the ‘Chains’ field for the calculations and an email address in the ‘Email’ field for large submissions. B) All jobs go through pre-analysis computations phase where the normal modes are calculated and some initial analyses are performed, and the URL here can be bookmarked for results that can be retrieved up to two weeks from the submission time. C) The pre-analysis page shows results such as the deformation energies of the lowest modes are provided, and the eigenvalues. Any further analysis on the given protein structure can be done by clicking the ‘Proceed to analyses’ button at the bottom of the page. The ‘Analysis’ page provides links to the ‘Atomic Displacement Analysis’ and ‘Mode Visualization and Vector Field Analysis’ and provides further opportunities to perform the ‘Single Overlap Analysis’ and Correlation Matrix Analysis’. For the ‘Single Overlap Analysis’, a PDB ID and its corresponding chain ID or a coordinate file of the same protein in a different conformational state can be provided in the fields laid out in the same format as the initial submission tab. D) The ‘Mode Visualization and Vector Field Analysis’ page provides a Jmol applet where the lowest six modes are animated. The vector-field of the vibrations can also be visualised by selecting the ‘Vectors’ box, while deselecting the ‘Vibrations’ box can stop the animation. The vector fields can also be downloaded. The ‘Correlation Matrix Result’ page provides the correlation matrix heatmap as a downloadable PDF file, along with the raw data in text format. In addition, a PyMOL script visualising the significant correlations is also available for download. The ‘Overlap results’ of the single overlap analysis provides plots of the squared inner product between the difference vector of the two conformations and the full set of normal modes of the starting structure. The cumulative overlap shows the coverage

of the normal modes of the starting structure in the transition. Moreover, the linear interpolation between the two conformations is visualised in Jmol. All the analysis results pages come with a brief description of the method and references, that can be viewed by expanding the ‘See more…” links.

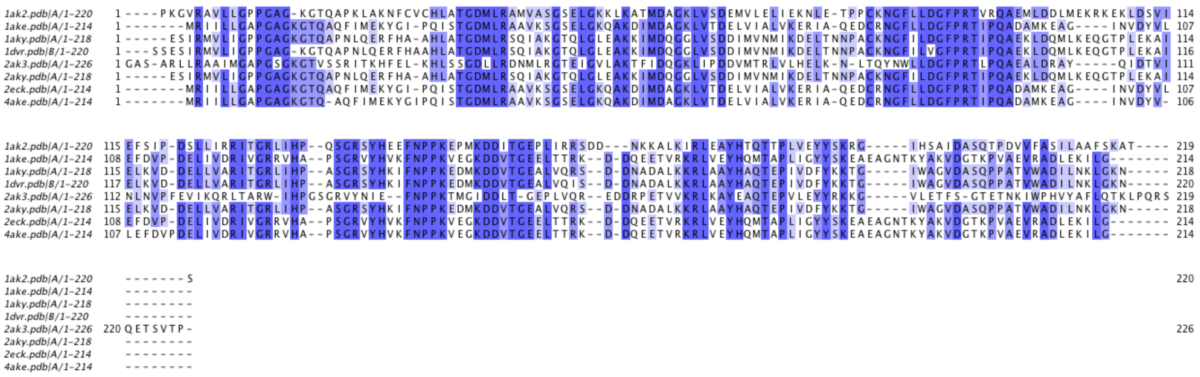

Figure S2. Sequence representation of the MUSTANG structural alignment of the eight Adenylate Kinases from case study 2 in different ligand bound states. The alignment consists of PDB sequences as described in Table 1 in the main text. The shading in blue represents the level of amino acid conservation in the alignment, from lighter (low) to darker (high).

c.1.2		c.1.8	
c.1.2.3_1 A	c.1.2.4_1 A	c.1.8.3_1 A	c.1.8.4_1 A
c.1.2.3_2 A	c.1.2.4_2 A	c.1.8.3_2 A	c.1.8.4_2 A
c.1.2.3_3 A	c.1.2.4_3 A	c.1.8.3_3 A	c.1.8.4_3 M
c.1.2.3_4 A	c.1.2.4_4 A	c.1.8.3_4 A	c.1.8.4_4 A
c.1.2.3_5 A	c.1.2.4_5 A	c.1.8.3_5 A	c.1.8.4_5 A

Table S1. SCOP Data set for TIM-barrel proteins fold case study 1. 20 structures are from 2 SCOP superfamilies, c.1.2 and c.1.8, and from each of these, 2 families are equally represented. The naming follows the SCOP ID, followed by the single letter chain ID.

	1ak2.pdb	1ake.pdb	1aky.pdb	1dvr.pdb	2ak3.pdb	2aky.pdb	2eck.pdb	4ake.pdb
1ak2.pdb	100	59.57	67.65	66.38	55.31	67.65	59.57	59.57
1ake.pdb	59.57	100	64.68	62.97	51.91	64.68	100	100
1aky.pdb	67.65	64.68	100	98.29	56.17	100	64.68	64.68
1dvr.pdb	66.38	62.97	98.29	100	56.17	98.29	62.97	62.97
2ak3.pdb	55.31	51.91	56.17	56.17	100	56.17	51.91	51.91
2aky.pdb	67.65	64.68	100	98.29	56.17	100	64.68	64.68
2eck.pdb	59.57	100	64.68	62.97	51.91	64.68	100	100
4ake.pdb	59.57	100	64.68	62.97	51.91	64.68	100	100

Table 2. Sequence similarity between the homologous Adenylate Kinases (case study 2). All values here are presented as percentages, with 100% reflecting complete similarity where two sequences likely to be from the same organism.
